# Ttn as a likely causal gene for QTL of alcohol preference on mouse chromosome 2

**DOI:** 10.1186/1471-2105-15-S10-P12

**Published:** 2014-09-29

**Authors:** Lishi Wang, Yan Jiao, Yue Huang, Beth Bennett, Robert W Williams, Dawei Li, Hongyu Zhao, Joel Gelernter, Henry R Kranzler, Lindsay A Farrer, Weikuan Gu

**Affiliations:** 1 Department of Orthopaedic Surgery & BME - Campbell Clinic and Pathology, University of Tennessee Health Science Center, Memphis, TN 38163, USA; 2 Department of Basic Medical Research, Inner Mongolia Medical University, Hohhot City, Inner Mongolia, 010110, China; 3 Department of Pharmacology, University of Colorado Denver, Aurora CO 80045-0508, USA; 4 Department of Anatomy and Neurobiology, University of Tennessee Health Science Center, Memphis, TN, 38163, USA; 5 Department of Psychiatry, School of Medicine, Yale University, New Haven, CT 06510, USA; 6 Department of Epidemiology and Public Health, School of Medicine, Yale University, New Haven, CT 06510, USA; 7 Department of Genetics, School of Medicine, Yale University, New Haven, CT 06510, USA; 8 VA Connecticut Healthcare Center, West Haven, CT 06516, USA; 9 Department of Neurobiology, Yale University School of Medicine, New Haven, CT 06510, USA; 10 Department of Psychiatry, University of Pennsylvania School of Medicine, Philadelphia, PA 19104, USA; 11 VISN 4 MIRECC, Philadelphia VAMC, Philadelphia, PA 19104, USA; 12 Departments of Medicine (Biomedical Genetics), Neurology, Ophthalmology, Genetics & Genomics, Biostatistics, and Epidemiology, Boston University Schools of Medicine and Public Health, Boston, MA 02118, USA

## Background

Many quantitative trait loci (QTL) influencing mouse model phenotypes for alcoholism have been mapped genetically. However, the gene(s) comprising the QTL (QTG) are largely unknown. In previous work, Bennett and colleagues created congenic strains carrying the DBA/2IBG (D2) region for alcohol preference (AP) on chromosome 2, on a C57BL/6IBG (B6) background [1]. Subsequently, interval specific congenic recombinant strains (ISCRS), in which the full D2 QTL region was broken into smaller, partially overlapping regions of introgression, were generated and tested. With information from two ISCRS, the QTL has been mapped onto mouse chromosome 2 (Chr2) in a region of 3.4Mb by using C57BL/6J (B6) x DBA/2J (D2) recombinant inbred (RI) strains as well as by using F2 populations. Several candidate genes, *Gad1*, *Atp5g3*, *Atf2*, *Sp3* and *Sp9*, have been evaluated but none of them has been confirmed for a definitive role in the regulation of the QTL of AP on Chr2 [2,3].

## Materials and methods

We have been searching candidate genes for this QTL intensively by using an integrative approach including: 1) bioinformatics tools to search potential functionally relevant genes of alcohol preference within the QTL region [4]; 2) searching for single nucleotide polymorphisms (SNPs) within the exons of every gene between B6 and D2 in the QTL region; 3) conducting real time PCR to examine the differentially expressed genes between B6 and alcohol preferred interval-specific congenic recombinant strains (ISCRS); and 4) analysis of the association of candidate genes in the human population.

## Results

Titin (Ttn) is known as a giant muscle protein expressed in the cardiac and skeletal muscles. However, its expression level in the tongue is known to be higher than that in the heart. We therefore investigated if Ttn plays a role in the regulation of AP. Our data indicated that 1) the expression level of Ttn in the less AP congenic strains is significantly higher than that in B6; 2) the expression of a Ttn probe in the BXD RI strains is negatively correlated to that of AP; 3) One SNP is up- and the other is down-stream of Ttn. The alcohol consumption of the B6 genotype is significantly higher than that of D2 genotype in the BXD RI strains, based on data from multiple reports using two-bottles of choice; and 4) the polymorphism of TTN in the human population is highly associated with alcoholism.

## Conclusions

We conclude that Ttn is likely a causal gene for the QTL on Ch2 for the AP.
